# Barriers to retention in methadone maintenance therapy among people who inject drugs in Bangkok, Thailand: a mixed-methods study

**DOI:** 10.1186/s12954-017-0189-3

**Published:** 2017-09-07

**Authors:** Kanna Hayashi, Lianping Ti, Prempreeda Pramoj Na Ayutthaya, Paisan Suwannawong, Karyn Kaplan, Will Small, Thomas Kerr

**Affiliations:** 1 British Columbia Centre for Excellence in HIV/AIDS, St. Paul’s Hospital, 608-1081 Burrard Street, Vancouver, BC V6Z 1Y6 Canada; 2 Faculty of Health Sciences, Simon Fraser University, Blusson Hall, Room 11300, 8888 University Drive, Burnaby, BC V5A 1S6 Canada; 3 Department of Medicine, Faculty of Medicine, University of British Columbia, 317-2194 Health Sciences Mall, Vancouver, BC V6T 1Z3 Canada; 4 International Reference Group on Transgender Women and HIV/AIDS, Global Forum on MSM & HIV, 436 14th Street, Suite 100, Oakland, CA 94612 USA; 5 Thai AIDS Treatment Action Group, 18/89 Vipawadee Rd., soi 40 Chatuchak, Bangkok, 10900 Thailand; 6 Asia Catalyst, 1270 Broadway, Suite 1109, New York, NY 10001 USA; 7 Research Scientist, BC Centre for Excellence in HIV/AIDS, St. Paul’s Hospital Chair in Substance Use Research and Assistant Professor, Faculty of Health Science, Simon Fraser University, 608-1081 Burrard Street, Vancouver, BC V6Z 1Y6 Canada

**Keywords:** Methadone maintenance therapy, Drug law enforcement, Injection drug use, Harm reduction, HIV, Thailand

## Abstract

**Background:**

Methadone maintenance therapy (MMT) is a mainstay for treating opioid use disorder and preventing and managing HIV among people who inject drugs (PWID). While previous research suggested low dosing of methadone and high rates of discontinuation of MMT among PWID in Thailand, little is known about patients’ lived experiences with MMT in this setting. Therefore, we conducted a mixed-methods study to examine barriers to retention in MMT among PWID in Bangkok, Thailand, with particular attention to methadone dosing.

**Methods:**

Bivariate statistics were used to analyze quantitative survey data collected from methadone-treated PWID between July and October 2011. Qualitative data collected through semi-structured interviews with 16 methadone-treated PWID between July 2011 and June 2012 were analyzed thematically, with a focus on individual-level, social-structural, and environmental barriers to accessing MMT.

**Results:**

Among 158 survey participants, a median dosage of methadone was 30 mg/day (interquartile range 20–50). Of these, 15.8% reported having acquired street methadone due to low prescribed dosages of methadone and 19.0% reported recent syringe sharing. Qualitative interview data indicated some methadone provider-related barriers, including discouraging patients from using methadone due to it being a Western medicine, difficulty negotiating higher doses of methadone, and abrupt dose reductions without patient consultation (involving the provision of non-medicated “syrup” in some cases). Social-structural and environmental barriers to optimal MMT access included intense police surveillance of methadone clinics; and frequent incarceration of PWID and a lack of access to methadone in prisons.

**Conclusions:**

Among our sample of methadone-treated PWID, methadone dosages were suboptimal according to the international guidelines. Poor adherence to international guidelines for opioid agonist therapies, aggressive law enforcement, and a lack of methadone in prisons need to be addressed to optimize MMT and reduce harms associated with untreated opioid use disorder in Thailand.

## Background

Opioid agonist therapies, including methadone maintenance therapy (MMT), are the mainstay for treating opioid use disorder and preventing and managing HIV among people who inject drugs (PWID) [1, 2]. However, coverage of this essential service remains very low in many low- and middle-income countries, including in Southeast Asia, where injection drug use involving opioids has been a key driver of HIV epidemics [3, 4]. The number of PWID receiving opioid agonist therapies has been reported to be as low as less than four per 100 PWID in Southeast Asia, while it was more than 60 per 100 PWID in Western Europe [3].

Moreover, while adherence to evidence-based treatment guidelines, including adequate dosing of methadone, has proven suboptimal in North America [5], there is limited information on how methadone is provided in Southeast Asia [4, 6]. Qualitative studies of methadone patients’ experiences in China and Ukraine identified some multi-level barriers to MMT, including service providers’ preference for abstinence-based treatment models, inflexible dosing regimens, fear of addiction to methadone, police harassment near treatment sites, and stigma, discrimination, and mistreatment by service providers [7, 8]. However, these experiences were not investigated alongside the methadone doses received by patients. Available data suggest that methadone doses are typically low in Asia [9]. For example, a 2010 population-level study in Wuhan, China, reported that 69% of patients were *prescribed* ≥ 60 mg/day of methadone; however, the majority (82%) *actually received* < 60 mg/day of methadone [10]. These findings indicate the importance of examining actual methadone doses received by patients and how methadone dosing influences patients’ experiences and treatment outcomes.

Thailand has been contending with a dual epidemic of opioid injection and HIV for decades [11, 12]. While the use of methadone for maintenance therapy was approved in 2000 and became covered through a universal healthcare scheme in 2008, available data suggest some clinics in Bangkok have continued to provide inadequate doses of methadone [6, 13]. In Bangkok, methadone treatment is primarily provided by the Bangkok Metropolitan Authority through its 17 public health centers, two hospitals, and one stand-alone clinic. A previous study documented that ongoing injection drug use was common among methadone-treated PWID in Bangkok, indicating a need for further research to identify gaps between internationally recommended best clinical practices and the actual implementation of MMT in this setting [14]. In particular, Thailand has traditionally relied on prohibition-based drug policy, which has involved aggressive drug law enforcement and mass incarceration of PWID [15]. Such policy environment would likely affect availability and accessibility of MMT, as some previous studies have indicated [15, 16]; however, details have not been fully examined. Therefore, we conducted a mixed-methods study to examine barriers to retention in MMT among methadone-treated PWID in Bangkok, with particular attention to methadone dosing.

## Methods

### Study design

Data were derived from the Mitsampan Community Research Project, a collaborative research effort involving the Mitsampan Harm Reduction Center (MSHRC; a peer-run drop-in centre in Bangkok, Thailand), Thai AIDS Treatment Action Group (Bangkok, Thailand), Chulalongkorn University (Bangkok, Thailand), and the British Columbia Centre for Excellence in HIV/AIDS/University of British Columbia (Vancouver, Canada). Launched in 2008, this serial cross-sectional mixed-methods study aimed to investigate drug-using behaviour, healthcare access, and drug-related harms among PWID in Bangkok [14]. For the present mixed-methods study, we adopted a simple triangulation design suggested by Creswell and Clark, in which quantitative and qualitative data collection was implemented around the same time [17]. The study was approved by the research ethics boards at Chulalongkorn University and the University of British Columbia/Providence Health Care.

Between July and October 2011, the research partners surveyed 440 PWID in Bangkok. Potential participants were recruited through peer outreach efforts (on the street, near MMT clinics, etc. all over Bangkok) and word-of-mouth and were invited to attend the MSHRC or O-Zone House (another drop-in centre in Bangkok) to participate in the study. Adults residing in Bangkok or in adjacent provinces who had injected drug(s) in the previous 6 months were eligible for participation. All participants provided informed consent and completed an interviewer-administered questionnaire eliciting a range of information, including socio-demographic characteristics, drug use patterns, and related exposures. Upon completion of the questionnaire, participants received a stipend of 350 Thai Baht (approximately US$12).

A qualitative arm of the project was implemented between July 2011 and June 2012, involving 48 semi-structured interviews with PWID in Bangkok. The overarching objectives of the qualitative arm were to explore PWID’s experiences with policing compulsory drug detention centres and access to healthcare (including methadone treatment). The study was informed by Rhodes’ Risk Environment Framework, which encourages consideration of individual, social-structural, and environmental drivers of drug-related harm [18]. In relation to healthcare access, it sought to understand barriers to and facilitators of optimal healthcare and how they influence health outcomes [19]. Potential participants were recruited face-to-face from the concurrent quantitative arm of the project as well as through peer-based outreach efforts and word-of-mouth and were invited to attend the MSHRC or O-Zone House in order to participate in the study. While the eligibility criteria were consistent with those of the quantitative arm, we prioritized the recruitment of individuals with relevant experiences (e.g., having accessed methadone treatment) and made efforts to attain balance in age, gender, and HIV serostatus.

Two bilingual Thai interviewers conducted interviews in Thai based on a semi-structured interview guide. With respect to MMT, the interview guide sought to elicit discussion of experiences with MMT including how participants felt about MMT and how they felt MMT could be improved. The interview guide was reviewed by local community research partners, which served to fine-tune the questions. Interviewers were also encouraged to employ additional questions and probes to explore each individual respondent’s experience. Throughout the data collection process, the research team discussed the content of interview data as well as the focus and direction of subsequent interviews. Data collection was continued until data reached a point of saturation. All interviews were conducted in private rooms at the MSHRC and O-Zone House, lasted between 40 and 90 min, and were audio-recorded. All participants provided informed consent and received a stipend of 450 Thai Baht (approximately US $15) upon completion of the qualitative interview.

### Statistical analysis

For the present analysis, the sample was restricted to those who reported having received methadone treatment in the previous 6 months and who had complete data on methadone dosages. First, we calculated the median daily dosage of methadone most recently received by the participants. Given a previous systematic review reporting that at least 60 mg/day of methadone predicts favourable treatment outcomes [20], the primary outcome of interest was a binary variable denoting receiving ≥ 60 mg/day vs. < 60 mg/day of methadone. We also had the secondary outcome, which was a binary variable comparing > median dose vs. ≤ median dose, in order to examine any differences in receiving higher or lower doses within our sample. Explanatory variables considered included age (per year older); gender (male vs. female); HIV status (positive vs. negative or unknown); daily injection of heroin, midazolam (a short-acting benzodiazepine), methadone, and methamphetamine, respectively; syringe sharing; having ever acquired street methadone because prescribed dosages of methadone were too low; and duration of methadone treatment (< 1 month vs. ≥ 1 month, < 6 months vs. ≥ 6 months, < 12 months vs. ≥ 12 months). All behavioural variables referred to the previous 6 months unless otherwise stated. We used the Pearson’s *X* ^2^ test (for categorical variables) and the Wilcoxon rank sum test (for continuous variables) to examine bivariate associations between the explanatory variables and the outcome. Fisher’s exact test was used when one or more of the cells contained expected values less than or equal to five. All *p* values were two-sided. All statistical analyses were performed using SAS software version 9.4 (SAS, Cary, NC).

### Qualitative analysis

The sample was restricted to those who received methadone treatment during the past 5 years. All audio-recorded interviews were transcribed verbatim in Thai and translated into English. The bilingual interviewers reviewed the translated transcripts for accuracy. Further, a native English-speaking proofreader with an excellent knowledge of both Thai and English also verified the English transcripts for grammatical accuracy and nuance by comparing the English transcripts with Thai transcripts and audio-files. All data were entered into Atlas.ti (version 6.2).

Data analysis was focused on identifying different types of barriers to MMT, and how these function to impede retention in MMT, and was informed by the aforementioned Risk Environment Framework [18]. The analysis was conducted inductively, employing a multi-step thematic analysis. On the first pass, KH created an initial set of codes. Subsequent reviews involved refining the codes and assigning data segments to categories with substantive input from other co-authors. The analysis considered the range and diversity of participants’ experiences, as well as the negative evidence in each category of experience. Finally, the data were grouped into three primary categories: overall perceptions of MMT, methadone provider-related barriers, and social-structural and environmental barriers.

## Results

### Statistical analysis

In total, 194 participants reported having received methadone in the past 6 months. Of those, 36 (18.6%) did not have data on methadone doses or duration and were excluded from the analysis. There were no significant differences between those excluded and included in the analysis in terms of demographic and drug use characteristics as well as HIV-seropositivity (all *p* > 0.05). As shown in Table 1, among 158 eligible participants, 27 (17.1%) were women. The median age was 38 years (interquartile range (IQR) 34–48). Almost three-quarters (72.8%) were receiving methadone for more than a year. The median daily dosage of methadone was 30 mg (IQR 20–50; minimum 5; maximum 80), and 16 (10.1%) received ≥ 60 mg/day. Nearly half (48.1%) reported daily injection of midazolam, 14.6% injected methadone daily, and 13.9% injected heroin daily. Further, 19.0% reported syringe sharing, and 15.8% reported having ever acquired street methadone because prescribed dosages of methadone were too low. In bivariate analyses, HIV positivity was significantly associated with receiving ≥ 60 mg/day of methadone (*p* = 0.015), whereas younger age was significantly associated with receiving > median dose (30 mg/day) of methadone (*p* = 0.027).

**Table 1:** Bivariate analyses of factors associated with methadone doses among 158 methadone-treated PWID in Thailand

Characteristic	Total *n* (%) 158 (100)	Methadone dosage^b^		*p* value	Methadone dosage^b^		*p* value
		≥ 60 mg/day*n* (%)16 (10.1)	< 60 mg/day*n* (%)142 (89.9)		> 30 mg/day*n* (%)78 (49.4)	≤ 30 mg/day*n* (%)80 (50.6)	
Age (median, IQR)	38 (34–48)	36 (34–44)	38 (34–48)	0.273	37 (33–46)	40 (35–51)	0.027
Male gender	131 (82.9)	13 (81.2)	118 (90.1)	0.739^c^	65 (83.3)	66 (82.5)	0.889
HIV-positive	30 (19.0)	7 (43.8)	23 (16.2)	0.015^c^	18 (23.1)	12 (15.0)	0.196
Daily heroin injection^d^	22 (13.9)	1 (6.3)	21 (14.8)	0.701^c^	11 (14.1)	11 (13.8)	0.949
Daily midazolam injection^d^	76 (48.1)	8 (50.0)	68 (47.9)	0.873	39 (50.0)	37 (46.3)	0.637
Daily methamphetamine injection^d^	7 (4.4)	0 (0.0)	7 (4.9)	N/A	4 (5.1)	3 (3.8)	0.718^c^
Daily methadone injection^d^	23 (14.6)	2 (12.5)	21 (14.8)	>0.999^c^	13 (16.7)	10 (12.5)	0.458
Syringe sharing^d^	30 (19.0)	2 (12.5)	28 (19.7)	0.738^c^	13 (16.7)	17 (21.3)	0.463
Ever acquired street methadone because prescribed dosages of methadone were too low	25 (15.8)	3 (18.8)	22 (15.5)	0.721^c^	16 (20.5)	9 (11.3)	0.111
Duration of methadone treatment^a^							
< 1 month	4 (2.5)	0 (0.0)	4 (2.8)	N/A	1 (1.3)	3 (3.8)	N/A
≥ 1 month, < 6 months	26 (16.5)	2 (12.5)	24 (16.9)		10 (12.8)	16 (20.0)	
≥ 6 months, < 12 months	11 (6.7)	0 (0.0)	11 (7.7)		6 (7.7)	5 (6.3)	
≥ 12 month	115 (72.8)	14 (87.5)	101 (71.1)		60 (76.9)	55 (68.8)	

*PWID* people who inject drugs, *IQR* interquartile range

^a^Numbers do not add up to 158 due to missing observations (*n* = 2)

^b^At the most recent time they received methadone

^c^Fisher’s exact test

^d^Refers to the previous 6 months

### Qualitative analysis

In total, 16 participants were included in the present analysis, including five women and eight HIV-positive individuals. Collectively, these participants accessed ten different methadone clinics in Bangkok during the past 5 years, which covered half of all 20 methadone clinics operating in Bangkok [13]. Table 2 summarizes the participants’ demographic characteristics and recent drug use patterns.

**Table 2:** Qualitative study sample characteristics (*n* = 16)

Characteristic	*n* (%)
Male gender	11 (68.8)
Age (median, IQR)	45 (36–50)
HIV-positive	8 (50.0)
Daily heroin injection^a^	4 (25.0)
Daily midazolam injection^a^	13 (81.3)
Daily methamphetamine injection^a^	0 (0.0)
Daily methadone injection^a^	2 (12.5)
Methadone dosage in mg/day^b^	
Median (IQR)	35 (25–50)
Minimum–maximum	12–80

*IQR* interquartile range

^a^Refers to the previous six months

^b^At the most recent time they received methadone

As shown below, we used verbal counting to highlight patterns in the data [21]. In doing so, we operationally defined “many” and “common” as something reported by half or more of the participants and “some” and “a few” as something reported by less than one-third of the participants. However, inferences of generalizability and statistical significance from these terms are discouraged. All names appearing below are pseudonyms.

### Overall perceptions of methadone treatment

Many participants negatively perceived MMT as they felt that it did not help them reduce or stop their use of illicit drugs. The majority of participants attributed the ineffectiveness of MMT to low dosages of methadone:

*The methadone level is not enough. But they say they’ve already given me a lot: 40mg per day. …It can’t control my body because I also use heroin.* (Somsak, male, age 36)

Another common source of negative perceptions against methadone was the fear of experiencing methadone withdrawals, which participants believed to be more intolerable than those of heroin. Some participants reported abrupt methadone interruptions due to incarceration, which further fuelled their fear of methadone withdrawals.

A few participants reported having been able to reduce their heroin use while on methadone; however, it was also reported that midazolam use (via injection), a short-acting benzodiazepine, was either initiated or continued to alleviate sleep disturbance while on methadone.

### Methadone provider-related barriers

Participant accounts revealed that the most commonly experienced barriers to accessing MMT stemmed from methadone providers’ negative attitudes towards methadone and patients who use drugs. A few participants reported that doctors had first discouraged them from initiating MMT, either because it is a Western medicine, or because the doctors were concerned that the individuals would become addicted to methadone:

*I told him* [i.e., a doctor who prescribed methadone] *that I wanted to take the treatment. Then, he advised me to go cold turkey and not to believe Western doctors. He said, “This drug is from the West. Westerners are tricking us.” He was quite anti-Western medicine.* (Somsak, male, age 36)

*The doctor didn’t want me to take it* [i.e., methadone] *because I could become deeply addicted to it, in addition to other drugs I had already been addicted to. He just gave me some pills so that I would just deal with the aches.* (Sompong, male, age 34)

Methadone providers’ negative attitudes towards methadone and patients who continue to use drugs most commonly manifested themselves when participants requested higher doses of methadone. Except for a few isolated cases, participants reported significant difficulty in negotiating higher doses, as they would be reprimanded for continuing to use drugs or “relying too much on methadone.” No participant reported that methadone providers examined whether ongoing drug use stemmed from inadequate dosing of methadone. Further, some participants reported experiencing an abrupt dose reduction without prior consultation. In some cases, this involved the provision of non-medicated ‘syrup’ that did not contain methadone.

*Some doctors give us just the syrup. I’ve had that before. It was just the syrup, without methadone in it. As soon as I took it, I just knew! If I take methadone, my symptoms will stop. But at that time, I was yawning so much. Then I saw another doctor. I told him about my symptoms and that I suspected that it was just the syrup. Then he gave me a new glass* [of methadone syrup]*. In 10-15 minutes, my symptoms, all the yawning and goose bumps, were gone. …Some doctors reduce the dose without telling us! How could they just reduce it? ...When this happened, I talked to the head doctor. He accused me of relying too much on methadone.* (Nan, female, age 47)

Participants’ narratives also indicated that a lack of trust characterized relationships between methadone providers and patients, which further undermined engagement with MMT. Some participants reported that, when they did not immediately leave clinics after receiving their methadone dose, clinic staff suspected them of dealing drugs and threaten to call the police. Peer harm reduction outreach workers who distributed sterile syringes at methadone clinics were also accused of promoting drug use.

In response, some participants reported conflicts with clinic staff. As a consequence, they were suspended from accessing the clinic and transferred to another one. These hostile relationships served to undermine the possibility of open communication between methadone providers and patients and resulted in methadone discontinuation in some cases.

### Social-structural and environmental barriers

Consistent with previous studies in other settings [8], some programmatic barriers to MMT were noted, including difficulty maintaining full-time employment, limited operating hours of clinics, limited take-home privileges, and difficulty travelling to clinics. An additional barrier that many participants referred to was the ready availability of illicit drugs in the local setting, where a previous study also reported significant increases in the street-level availability of illicit drugs despite the intensified police crackdowns [22]. Combined with low-dose methadone, this appeared to pose challenges with staying on methadone and not using illicit drugs. In response to these dynamics, some participants used methadone only when they could not afford illicit opiates, as opposed to taking it as maintenance therapy.

Some participants also witnessed drug-dealing activities at methadone clinics and identified encountering their drug-using peers at clinics as something that could trigger relapse into drug use. Drug dealing at clinics also precipitated some police surveillance activities in the vicinity of clinics. Although participants felt safe inside clinics (as long as they did not interact with drug dealers), police harassment, and violence around clinics provoked fear and discouraged some from accessing MMT.

Further, because incarceration rates were high among PWID in this setting, and methadone was not available in prisons, some participants described incarceration as a major reason for methadone discontinuation, including a repeating cycle of interruption and re-initiation.

I: How long can you stay on the methadone treatment?

P: It’s periodic. If I get arrested, I have to stop my methadone treatment, right? After I get out of prison, I start taking it again. It’s been like that.

I: Going on and off. How many times have you been arrested?

*P: Around six times. Each time, about nine months in prison.* (Ball, male, age 42)

## Discussion

The median methadone dose (30 mg/day) among our sample of methadone-treated PWID was far below the internationally recommended maintenance dosage range (60–120 mg/day) [23]. Many participants also reported difficulty negotiating dose adjustments based on their individual needs. Low and inflexible methadone dosing is known to predict suboptimal treatment outcomes [20, 24], and challenges with dose adjustments have also been documented as a key barrier in other settings [8]. Not surprisingly, the prevalence of ongoing injection drug use and syringe sharing was also high among our sample, indicating that inadequate dosing significantly compromised potential HIV prevention benefits of MMT in this setting.

The finding that HIV-positive PWID were more likely to receive ≥ 60 mg/day of methadone suggests that some MMT providers may have appropriately increased the dose of methadone due to the interaction with antiretroviral treatments [23]. It is imperative to ensure that all HIV-positive PWID receive an optimal dose of methadone given that MMT retention contributes to the optimization of antiretroviral treatments [25]. In particular, MMT providers should ensure open communications with patients regarding HIV status and treatment. In the quantitative analysis, we also found that younger PWID were more likely to receive higher doses of methadone. However, issues related to age did not emerge from qualitative interviews. Future research should investigate and address the potential age difference in methadone doses.

Low dosing of methadone is common in other Asian countries, including China [6, 10], Indonesia [6], and Malaysia [26]. A qualitative investigation of methadone service providers in China documented that a lack of understanding of dosage management and harm reduction, and poor communication with patients resulted in low acceptance of MMT and the tendency to prescribe low dosages [27]. Our findings echo these observations and raise ethical concerns given the reported instances of abrupt dose reduction without appropriate patient consultation. Collectively, these findings indicate the importance of educating service providers about MMT and setting clear evidence-based therapeutic guidelines. Given some concerns that methadone is a western medicine, such education may best be delivered by local experts. Also, adding combination buprenorphine/naloxone, another evidence-based agent for opioid agonist therapy, to treatment options will address some safety concerns regarding methadone as expressed elsewhere [28, 29]. As recommended by health authorities in a range of other settings, buprenorphine/naloxone should be considered in this setting [29].

Participant narratives also indicated that aggressive drug law enforcement disrupted availability and accessibility of MMT. Police interference of MMT has been well documented in this setting [15, 16] and elsewhere [30]. While some efforts to sensitize police officers to harm reduction services for PWID have been initiated, these need to be scaled up and sustained [31]. Further, unless drug policies that heavily criminalize personal use of illicit drugs are eliminated, PWID continue to be at high risk of incarceration. Although the World Health Organization recommends the provision of opioid agonist therapies in correctional settings as a minimum standard [23], a recent report documented that coverage of such therapies remains suboptimal in many countries, with only < 1% of prisoners in need of treatment were able to access it [32]. Thailand is not an exception, and methadone remains unavailable in prisons even though the incarceration rate among PWID is extremely high, and incarceration has been associated with rapid spread of HIV infection in this setting [33]. Importantly, our findings suggest that fear of methadone withdrawal was also fuelled by the lack of methadone in prisons. As many in the international scientific community have repeatedly called for, the incarceration of PWID needs to be reduced through appropriate drug policy reform, and voluntary opioid agonist therapies need to be made available for those prisoners who require such treatment [32, 34].

Added value of this study lies in the unique evidence generated through its focus on PWID in Bangkok and its mixed-methods study design, which illustrated how inadequate dosing of methadone served to compromise the relationships between patients and service providers and the potential benefits of MMT. However, this study also has several limitations. First, as the sample for the quantitative study was not recruited randomly, or did not include non-PWID, our findings may not be generalizable to all Thai people who use drugs. Specifically, we did not record exact places or channels through which participants were recruited. Therefore, there may be unmeasured geographical differences. Also, our qualitative study findings were based on interviews with PWID who received methadone at ten different clinics during the previous 5 years. Therefore, experiences and views of non-PWID or PWID who accessed other methadone clinics were not included, and the transferability of the findings may be limited. Second, self-reported data may be subjected to reporting biases. Lastly, the low count of participants receiving ≥ 60 mg/day of methadone affected the statistical power of some bivariate analyses; however, we feel that the mixed-method design employed is the strength of our study, which served to enhance the integrity of our data.

## Conclusions

In sum, among our sample of methadone-treated PWID in Bangkok, methadone doses appeared too low to confer the maximum clinical benefit. Poor adherence to international clinical guidelines, aggressive law enforcement, and a lack of methadone in prisons need to be addressed to optimize opioid agonist therapies and reduce harms associated with untreated opioid use disorder in this setting.

## Abbreviations

MMT: Methadone maintenance therapy
MSHRC: Mitsampan Harm Reduction Center
PWID: People who inject drugs
